# Beyond the menu: how student agency, cultural acceptability, and equity shape realized sustainability in school food environments

**DOI:** 10.3389/fnut.2026.1956116

**Published:** 2026-09-07

**Authors:** Lu Xing, Xueqi Wu

**Affiliations:** 1 School of Preschool Education, Changsha Normal University, Changsha, China; 2 School of Education, Faculty of Social Sciences and Leisure Management, Taylor's University, Subang Jaya, Malaysia; 3 Taylor's Culinary Institute, Faculty of Social Sciences and Leisure Management, Taylor's University, Subang Jaya, Malaysia

**Keywords:** adolescents, children, cultural acceptability, equity, food waste, school food environment, student agency, sustainable diets

## Abstract

School food environments influence dietary intake, nutritional inequities, food habits, and exposure to sustainable eating during childhood. A nutritionally adequate, lower-impact meal yields public-health and environmental value only when pupils can access, select, consume, and accept it without disproportionate burden or waste. This structured Mini Review asks how student agency, cultural acceptability, and equity-related conditions shape the translation of sustainable school-food provision into selection, intake, waste, lived experience, and distributional outcomes. Web of Science and Scopus searches returned 1,521 records and 871 unique records. Rule-based prioritization and author-verified source assessment of 85 publications produced a 25-publication analytic set representing 23 study families: 13 Direct-child, two Direct-caregiver, five Direct-service/program, and five Adjacent publications. Study quality was appraised against the strongest available source using design-specific Mixed Methods Appraisal Tool criteria or adapted model-credibility domains; relative support was synthesized at study-family level. Sensory fit showed Moderate support for liking/acceptance only. Menu reform, waste feedback, education/information, agency, affordability/governance, and lower- and middle-income country transferability had Limited support, while identity/commensality and digital influence remained Very limited. In a multicountry non-randomized study, service-level plate-waste tracking was associated with lower measured waste; educational studies reported mixed or null outcomes, precluding comparative causal claims. Evidence for religion, migration, disability, commensality, and decision authority remained sparse. The framework changes the unit of evaluation to a provision–access/selection–intake–waste pathway with a cross-cutting equity audit and identifies governance, cultural recognition, and implementation capacity as conditions of realized sustainability.

## 1 Introduction

School food environments are an urgent public-health setting because children may consume a substantial share of weekday food at school, while menus, prices, service routines, and dining conditions shape nutrient intake, dietary inequities, and repeated food habits. School meals can support healthy growth and learning, normalize diverse foods, and reduce financial barriers; poor-quality or socially unacceptable provision can instead suppress participation, displace intake, or increase waste. These health consequences are inseparable from sustainability because procurement, menu composition, and discarded food determine whether environmental improvements reach children in an edible and equitable form (1–4). Long-term growth, cardiometabolic, educational, and life-course outcomes were rarely measured in the pathway evidence reviewed here.

Reviews have mapped environmental school-food interventions, sustainable healthy-diet programs for primary pupils, and sustainability-oriented nutrition education (1–3). A Cochrane protocol, adolescent reviews, and bibliometric work further delimit school-food interventions, perceptions, and publication patterns (4–7). These syntheses mainly organize evidence by intervention type, effectiveness outcome, perception, or research structure. They do not make the translation from sustainable provision to realized practice the primary object of review, nor do they jointly evaluate whether agency, cultural fit, and equity change selection, intake, waste, and the distribution of benefit.

This Mini Review therefore asks: **How do student agency, cultural acceptability, and equity-related conditions shape the translation of sustainable school-food provision into realized selection, intake, waste, lived experience, and distributional outcomes among children and adolescents, and what is the relative support for each pathway within the focused evidence set?** Its central contribution is a provision-to-practice framework that distinguishes what institutions offer from what pupils can access and choose, what they consume or discard, and who benefits. This distinction positions child nutrition, environmental performance, cultural recognition, and equity as linked implementation outcomes rather than parallel policy aspirations.

## 2 Methods

### 2.1 Review design, scope, and outcomes

**Figure 1:**
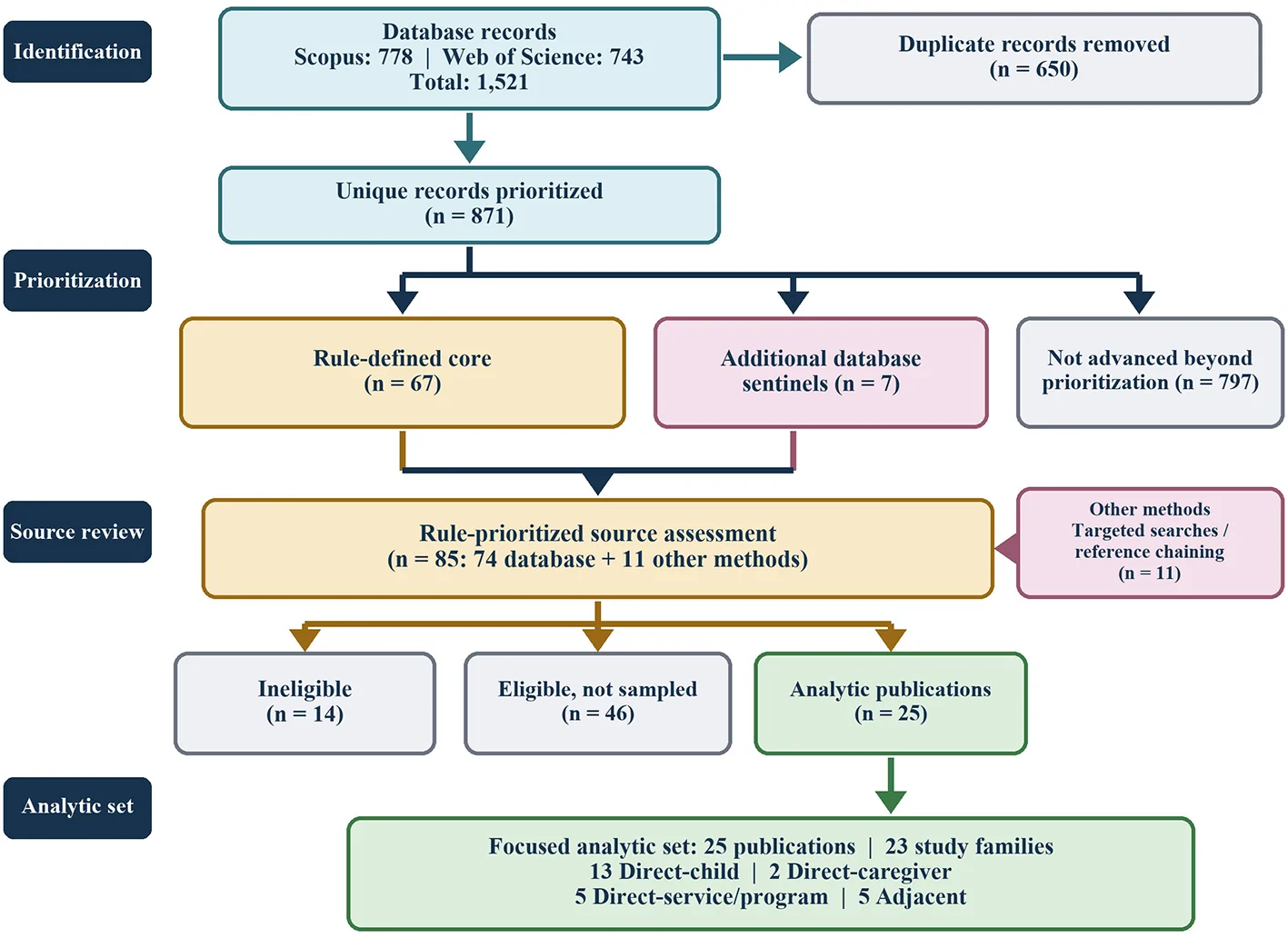
PRISMA-informed identification and analytic-sampling flow.

We conducted a non-exhaustive, rule-prioritized focused Mini Review with transparent identification, criterion-based sampling, and source-bounded appraisal, reported in a PRISMA-informed identification and analytic-sampling flow (Figure 1) (8). Eligible populations were children and adolescents aged approximately 5–18 years. Eligible settings comprised institutionally governed school meals, cafeterias, food services, menus, procurement, pricing or subsidy, dining routines, plate-waste systems, and provision-linked education. Direct evidence required an explicit environmental or sustainable-food-system exposure or outcome—such as food-waste reduction or measurement, plant-forward or lower-impact menus, sustainable procurement, greenhouse-gas reduction, or whole-food utilization—linked to an outcome in the same analysis. The respondent and outcome level was then recorded as Direct-child, Direct-caregiver, or Direct-service/program. Adjacent evidence measured a target mechanism in an institutionally governed school-food setting without establishing an environmental exposure or outcome.

The primary realized-practice outcomes were food selection, intake or consumption, and plate waste. Secondary outcomes were sensory acceptance, satisfaction, participation, stigma, perceived agency, cultural recognition, and distribution across pupil or school groups. Modeled nutrition, environmental impact, and cost described provision potential rather than realized child outcomes. Reviews and protocols were used to position the review but were not counted as pathway-effect evidence.

### 2.2 Information sources and search strategy

Web of Science Core Collection and Scopus were searched for journal articles and reviews published from 2000 through 30 July 2026. Eligibility required an English title and abstract that permitted record-level screening; full-text language was not restricted. Accordingly, Quezada-Figueroa et al. (9) was eligible because its indexed record supplied an English title and abstract, although the full article was in Spanish and was assessed from the original report. Database search blocks combined school meals, canteens, cafeterias, procurement, food service, and dining environments with sustainability, food waste, plant-forward provision, lower-impact sourcing, and greenhouse-gas emissions. A rule map then identified social-cultural, agency, equity, and digital signals during prioritization. Database-specific full strings, interface limits, retrieval dates, source-file hashes, and export counts are reproduced in Supplementary Table S1. Seven database records outside the strict lexical core and 11 records identified through reference chaining or targeted pathway searches formed an 18-publication coverage set.

### 2.3 Screening, classification, and study families

Database exports were deduplicated by DOI, accession identifier, and normalized title. The authors developed a deterministic rule map identifying explicit social-cultural, agency, equity, and realized-practice signals. Two separate, rule-constrained AI-assisted title-and-abstract prioritization passes were generated in OpenAI Codex (OpenAI; GPT-5, model identifier gpt-5, accessed July–August 2026; https://openai.com/codex/). Each pass returned provisional include, exclude, or uncertain labels with reason codes. These outputs served only as non-binding sorting and audit aids; they were not independent human screens and did not determine eligibility. Agreement between the passes and model-vote reconciliation were not used. The author-defined deterministic rule map was applied separately to all 871 records and advanced 67 core and seven additional database records; the remaining 797 records were not advanced and are not presented as eligibility exclusions. Eleven records identified through other methods were added for targeted pathway coverage. Both authors reviewed the final 85-record source ledger and cross-checked the core information for all 25 included publications against the strongest accessible sources. Formal title-and-abstract screening of all 871 records was not independently duplicated by human reviewers. Final eligibility, extraction, appraisal, evidence-strength, and synthesis decisions were made and verified by the authors; discrepancies identified during verification were resolved through author discussion rather than model voting.

Of the 85 source-assessed publications, 14 were ineligible and 71 were eligible. For this focused review, the ordered sampling algorithm was fixed after corpus mapping and before the 25-publication analytic set was locked; it was not a prospectively registered protocol. The algorithm: (1) retained at most one publication per study family unless linked reports supplied non-overlapping effect and implementation evidence; (2) prioritized Direct-child evidence; (3) prioritized observed selection, intake, or waste over self-reported, hypothetical, qualitative, or modeled outcomes; (4) used Direct-service/program, Direct-caregiver, and then Adjacent evidence to fill mechanisms not represented at child level; (5) preferred a full-text, complete appraisal when evidential contribution was otherwise comparable; and (6) retained null findings while maximizing design, geographic, resource-context, and pathway variation. No fixed pathway quota was used. Sampling stopped at the article-level cap of 25 fixed before final ranking to keep the Mini Review focused; ties were resolved in favor of an independent study family and then broader geographic or resource-context coverage. Supplementary Table S1 reports the superseding 85-row ledger, row-level sampling bases, exclusion reasons, source-access limits, and coverage sensitivity for all 46 eligible non-sampled publications.

Directness was recorded at the level supported by the respondent and outcome, not as a quality score. Direct-child required an explicit sustainability exposure or outcome and a measured child outcome in the same analysis. Direct-caregiver denoted parent or caregiver choices, willingness, or perceptions and was not treated as realized child behavior. Direct-service/program denoted modeled menus, service operations, staff or stakeholder outcomes, or program-level performance without a measured realized child outcome. Adjacent evidence informed a target mechanism but did not demonstrate an environmental exposure or outcome. Ambiguous studies were assigned the lowest directness level supported by the measured respondent and outcome. The final set comprised 13 Direct-child, two Direct-caregiver, five Direct-service/program, and five Adjacent publications. Staff-only evidence did not contribute to child-outcome strength. Publications sharing participants, intervention sites, or project infrastructure were grouped as one study family when judging replication. The linked OPTIMAT effect and implementation publications were synthesized as one intervention family, and Dijon parent-willingness and child-liking studies were treated as different respondent units within one municipal context. A probable overlapping Italian nine-school publication was held outside the analytic set, and independence was not assumed.

### 2.4 Quality appraisal and evidence-strength synthesis

Empirical studies were appraised against the strongest source available with design-specific 2018 Mixed Methods Appraisal Tool domains (10). No numeric total score was calculated; domain judgments were reported as Yes, No, or Cannot tell. A full-text source permitted a finalized design-specific appraisal, but did not imply low risk of bias or complete reporting. Source-limited appraisals based on an official abstract or landing record were marked partial, and every unverifiable domain remained Cannot tell rather than being treated as equivalent to a full-text appraisal. The Brazilian Amazon journal-supplement abstract was retained as an explicit geographic exception and was not assigned a finalized MMAT appraisal. Optimization or decision-model studies were assessed with an adapted domain set covering problem definition, data inputs, assumptions, validation, uncertainty, transparency, and applicability, informed by ISPOR–SMDM model-credibility principles (11). Table 1 and Supplementary Table S1 report the source available and appraisal completeness for every analytic publication, together with country, setting, sample, design, follow-up, outcome basis, and study family.

**Table 1:** Study characteristics, evidence level, outcome type, source availability, and quality appraisal of pathway publications.

Study	Country/setting	Sample, design, and follow-up	Evidence level and outcome type	Main result, limitation, source/appraisal
André et al. (12)	Sweden; six schools	Routine meal records and pupil surveys (school *n* = 164–857); controlled non-randomized intervention; 7+7 weeks.	Direct-child; observed consumption/waste + pupil-reported satisfaction.	A 20% lower-GHG menu was not associated with a significant short-term decline; non-randomized schools. Appraisal: MMAT non-randomized (D1:N, D2:Y, D3:N, D4:N, D5:Y; no total score). Source/appraisal: full text—complete appraisal.
Eustachio Colombo et al. (15)	Ghana; national menu model	No human sample; five linear-programming scenarios; follow-up not applicable.	Direct-service/program; modeled provision.	Nutrient/cost solutions increased some environmental pressures; assumptions and implementation untested. Appraisal: ISPOR–SMDM model (D1:Y, D2:Y, D3:Y, D4:Y, D5:CT, D6:CT, D7:Y, D8:Y; no total score). Source/appraisal: source-limited—partial appraisal.
Caso et al. (23)	Country not stated; online parent survey	617 parents; randomized hypothetical menu-choice experiment; no follow-up.	Direct-caregiver; hypothetical caregiver choice.	A norm message was associated with more lower-impact hypothetical choices with education heterogeneity; hypothetical outcome. Appraisal: MMAT randomized controlled trial (D1:CT, D2:CT, D3:Y, D4:Y, D5:Y; no total score). Source/appraisal: source-limited—partial appraisal.
Piochi et al. (17)	Italy; nine primary schools	630 children aged 7–11; cross-sectional convenience sample; no follow-up.	Direct-child; child self-report.	Neophobia predicted poorer liking and more reported waste; selection and reporting bias. Appraisal: MMAT quantitative descriptive (D1:Y, D2:N, D3:CT, D4:CT, D5:N; no total score). Source/appraisal: full text—complete appraisal.
Piras et al. (26)	Italy; 20 primary classes	420 pupils; controlled class-level education study; immediate and later assessment.	Direct-child; child self-report.	Short-term changes were not sustained; allocation and self-report limitations. Appraisal: MMAT randomized controlled trial (D1:Y, D2:Y, D3:N, D4:N, D5:Y; no total score). Source/appraisal: full text—complete appraisal.
Hennchen et al. (30)	Germany; 25 secondary schools	3,015 pupil surveys plus six stakeholder events; cross-sectional mixed methods.	Direct-service/program; pupil survey + qualitative program process.	Participation, sustainability, inclusion, and capacity produced tensions; no longitudinal outcomes. Appraisal: MMAT mixed methods (D1:Y, D2:Y, D3:Y, D4:CT, D5:N; no total score). Source/appraisal: full text—complete appraisal.
Serebrennikov et al. (27)	United States; three schools	135 consented/98 analyzed; cluster RCT; six weeks.	Adjacent; observed selection/waste.	No significant selection or waste effect; attrition and unclear cluster adjustment. Appraisal: MMAT randomized controlled trial (D1:Y, D2:Y, D3:N, D4:CT, D5:Y; no total score). Source/appraisal: full text—complete appraisal.
Eustachio Colombo et al. (13)	Sweden; three schools	Aggregate kitchens; 272/270 survey responses; interrupted time series; 4+4 weeks.	Direct-child; observed service/child outcomes + modeled impacts.	GHG fell 40% and cost 11% without significant behavioral decline; short nonrandomized study. Appraisal: MMAT non-randomized (D1:N, D2:Y, D3:Y, D4:N, D5:Y; no total score). Source/appraisal: full text—complete appraisal.
Eustachio Colombo et al. (14)	Sweden; three OPTIMAT schools	29 pupils and 13 staff; qualitative focus groups; post-intervention only.	Direct-child; qualitative pupil/staff experience.	Sensory, peer, leadership, and kitchen-capacity barriers emerged; one linked intervention family. Appraisal: MMAT qualitative (D1:Y, D2:Y, D3:Y, D4:Y, D5:Y; no total score). Source/appraisal: full text—complete appraisal.
Dahmani et al. (18)	France; Dijon public canteens	909 parents representing 1,261 children; optional section: 668/921; cross-sectional.	Direct-caregiver; caregiver-reported willingness.	Willingness varied by motives and household factors; opt-in parent report, not child behavior. Appraisal: MMAT quantitative descriptive (D1:Y, D2:N, D3:Y, D4:N, D5:Y; no total score). Source/appraisal: source-limited—partial appraisal.
Proietti et al. (16)	Kenya; Nairobi informal-settlement school	No human sample; menu model using five underutilized species; follow-up not applicable.	Adjacent; modeled provision.	Model broadened affordable ingredients; environmental impact, feasibility, and acceptance untested. Appraisal: ISPOR–SMDM model (D1:Y, D2:N, D3:Y, D4:Y, D5:CT, D6:CT, D7:N, D8:N; no total score). Source/appraisal: full text—complete appraisal.
Mahdi et al. (31)	United Kingdom; seven secondary schools	42 pupils; 38 food records/190 person-days; 35 focus-group participants; one week.	Direct-child; observed food records + qualitative pupil experience.	Price, payment, taste, and availability constrained choice; small participatory sample. Appraisal: MMAT mixed methods (D1:Y, D2:Y, D3:Y, D4:Y, D5:Y; no total score). Source/appraisal: full text—complete appraisal.
Humbelino de Melo et al. (21)	Brazil; one Amazonas riverside school	37 ninth-grade pupils; menu audit and questionnaire; no follow-up; supplement abstract.	Direct-child; menu audit + child self-report.	Menus underused biodiversity and 57% disliked provision; source details insufficient for finalized MMAT. Appraisal: Source-boundary abstract (D1:CT, D2:CT, D3:CT, D4:CT, D5:CT; no total score). Source/appraisal: journal-supplement abstract only—no finalized MMAT appraisal.
Marty et al. (19)	France; 38 Dijon canteens	208,985 votes across 125 dishes; repeated routine ratings over one school year.	Direct-child; child-reported connected liking ratings.	Vegetarian and nonvegetarian liking was similar overall; unique pupils and intake unreported. Appraisal: MMAT quantitative descriptive (D1:Y, D2:N, D3:Y, D4:N, D5:Y; no total score). Source/appraisal: source-limited—partial appraisal.
Kesa et al. (22)	South Africa; 25 primary schools	10-day diaries, 75 staff interviews, 25 learner groups; mixed methods.	Direct-child; staff-reported waste diaries + qualitative pupil/staff evidence.	Familiarity, preparation, and service shaped sorghum acceptance; learner denominator unreported. Appraisal: MMAT mixed methods (D1:Y, D2:Y, D3:Y, D4:CT, D5:N; no total score). Source/appraisal: full text—complete appraisal.
Zanoni et al. (20)	Twelve European countries	2,324 children aged 9–15; cross-sectional sensory/questionnaire study.	Direct-child; child self-report.	Liking related to lower reported waste; common-method and cross-sectional limitations. Appraisal: MMAT quantitative descriptive (D1:Y, D2:N, D3:CT, D4:CT, D5:N; no total score). Source/appraisal: source-limited—partial appraisal.
Mejía Toro et al. (34)	New Zealand; national lunch program	Workshops with 43 participants and 11 officials; participatory evaluation; no child follow-up.	Direct-service/program; stakeholder program evaluation.	Waste and procurement benefits coexisted with continuity risks; stakeholder judgments, no child outcomes. Appraisal: MMAT mixed methods (D1:Y, D2:Y, D3:Y, D4:Y, D5:N; no total score). Source/appraisal: full text—complete appraisal.
Zhao et al. (32)	United States; three states	47 SNAP-eligible pupils aged 9–13; qualitative interviews; no follow-up.	Adjacent; qualitative pupil-reported mechanism.	Pupils linked waste to quality, rules, and limited control; small self-selected sample. Appraisal: MMAT qualitative (D1:Y, D2:Y, D3:Y, D4:Y, D5:Y; no total score). Source/appraisal: full text—complete appraisal.
Prescott et al. (25)	United States; two middle schools	268 curriculum and 426 campaign pupils; non-randomized mixed methods; six points/five months.	Direct-child; observed selection/waste + pupil self-report.	Vegetable waste declined, but baseline differences weakened attribution. Appraisal: MMAT non-randomized (D1:N, D2:Y, D3:N, D4:N, D5:N; no total score). Source/appraisal: full text—complete appraisal.
Blondin et al. (33)	United States; ten urban schools	85 pupils, 86 parents, 44 teachers, 10 cafeteria managers, and 10 principals; qualitative study.	Adjacent; qualitative multistakeholder mechanism.	Universal access still produced surplus and service barriers; no environmental or causal measure. Appraisal: MMAT qualitative (D1:Y, D2:Y, D3:Y, D4:Y, D5:Y; no total score). Source/appraisal: full text—complete appraisal.
Jones et al. (28)	England; 30 primary schools	Stage 1 *n* = 1,435; stage 2 *n* = 1,463; repeated cross-sections; 18–24 months; no control.	Direct-child; pupil-reported intake + program delivery.	Stronger delivery related to fruit/vegetable intake; uncontrolled, partly self-reported evaluation. Appraisal: MMAT non-randomized (D1:N, D2:CT, D3:Y, D4:N, D5:Y; no total score). Source/appraisal: source-limited—partial appraisal.
Onuorah et al. (35)	Global; school-meal programs	Program-survey analysis, 2019–2024; contributing denominator not reported.	Direct-service/program; program-reported outcomes.	Plant-based and waste practices were common; program-level data did not establish child effects. Appraisal: MMAT quantitative descriptive (D1:Y, D2:CT, D3:Y, D4:CT, D5:CT; no total score). Source/appraisal: full text—complete appraisal.
Quezada-Figueroa et al. (9)	Chile; central-southern school canteens	33 female food handlers; qualitative focus groups; no follow-up.	Adjacent; qualitative food-handler perceptions.	Handlers identified cultural and operational causes; pupils and environmental outcomes were not measured. Appraisal: MMAT qualitative (D1:Y, D2:Y, D3:Y, D4:Y, D5:Y; no total score). Source/appraisal: full text—complete appraisal.
Sundin et al. (24)	Austria, Germany, Sweden; 24 schools	Tracker 12 canteens, pedagogic meals five, workshops 11; non-randomized; up to 15 months.	Direct-service/program; observed service waste + modeled impacts.	Tracker waste fell 17%; pedagogic meals/workshops were null; voluntary sites and changing baselines. Appraisal: MMAT non-randomized (D1:N, D2:Y, D3:CT, D4:N, D5:CT; no total score). Source/appraisal: source-limited—partial appraisal.
Goulart et al. (29)	Brazil; one southern school	48 children (mean age 8.9) plus mothers; co-design/sensory comparison; no follow-up.	Direct-child; observed child sensory ratings + qualitative caregiver responses.	Child/cook recipes outperformed ChatGPT recipe; exploratory one-school comparison. Appraisal: MMAT mixed methods (D1:Y, D2:Y, D3:Y, D4:CT, D5:Y; no total score). Source/appraisal: full text—complete appraisal.

Direct-child, Direct-caregiver, Direct-service/program, and Adjacent denote the respondent/outcome level of relevance to the provision-to-practice question, not study quality. Outcome types are tagged as observed, self-reported, hypothetical, modeled, qualitative, or program-reported. Full text—complete means that a full report supported a finalized design-specific appraisal; it does not imply low risk of bias. Source-limited—partial means that unverifiable domains were retained as CT. Humbelino de Melo et al. (21) is a journal-supplement abstract and has no finalized MMAT appraisal. For empirical studies, MMAT D1–D5 are the five design-specific criteria, with exact wording in Supplementary Table S1. For model studies, D1–D8 denote problem/context, structure/assumptions, input data, parameter estimation, internal verification, external validation, uncertainty/scenario analysis, and transparency/reproducibility. Linked publications were grouped as study families when judging replication. CT, cannot tell; LMIC, lower- and middle-income country.

Within the criterion-sampled analytic set, pathway narrative relative support was judged Moderate, Limited, or Very limited; these labels are not GRADE certainty ratings and no numeric weights or additive score were used. Ratings were anchored to independent study families rather than publication counts. The ordered judgment first considered directness at the measured outcome level, then source and design-appraisal limitations, consistency across independent families including null findings, observed vs. self-reported, hypothetical, qualitative, or modeled outcomes, and follow-up. Moderate required convergent direct findings across at least two independent families with measured child or service outcomes and no major unresolved inconsistency. Limited indicated a credible signal constrained by nonrandomized design, self-report, short follow-up, source limitations, or few settings. Very limited comprised exploratory, qualitative, modeling-only, or Adjacent evidence without a demonstrated realized-sustainability outcome. Coverage sensitivity mapped all 46 eligible non-sampled publications by pathway, design role, family, and likely effect on the category; none supplied independent higher-certainty evidence that justified an upward rating. Heterogeneity precluded meta-analysis and formal publication-bias tests, so contradictory and null findings were retained and publication-bias risk was considered qualitatively.

## 3 Results and pathway synthesis

### 3.1 Study selection and evidence profile

The searches returned 778 Scopus and 743 Web of Science records, representing 871 unique records after 650 duplicates were removed. Rule prioritization advanced 67 core and seven additional database records; 797 were not advanced. Reference chaining and targeted searches identified 11 further records, giving 85 source-assessed publications. Fourteen were ineligible, 46 were eligible but not sampled, and 25 publications representing 23 study families were retained (Figure 1): 13 Direct-child, two Direct-caregiver, five Direct-service/program, and five Adjacent. Seventeen publications were appraised from full reports, seven had source-limited partial appraisals, and one journal-supplement abstract had no finalized appraisal. The sample comprised controlled and randomized interventions, longitudinal and observational child studies, qualitative and participatory research, menu models, and program-level analyses. Objective selection, intake, or waste outcomes remained less common than modeled provision, liking, intention, self-report, or implementation evidence. Europe and other high-income settings predominated, but Ghana, Kenya, the Brazilian Amazon, South Africa, and Chile contributed modeling, observational, mixed-methods, or qualitative evidence.

**Figure 2:**
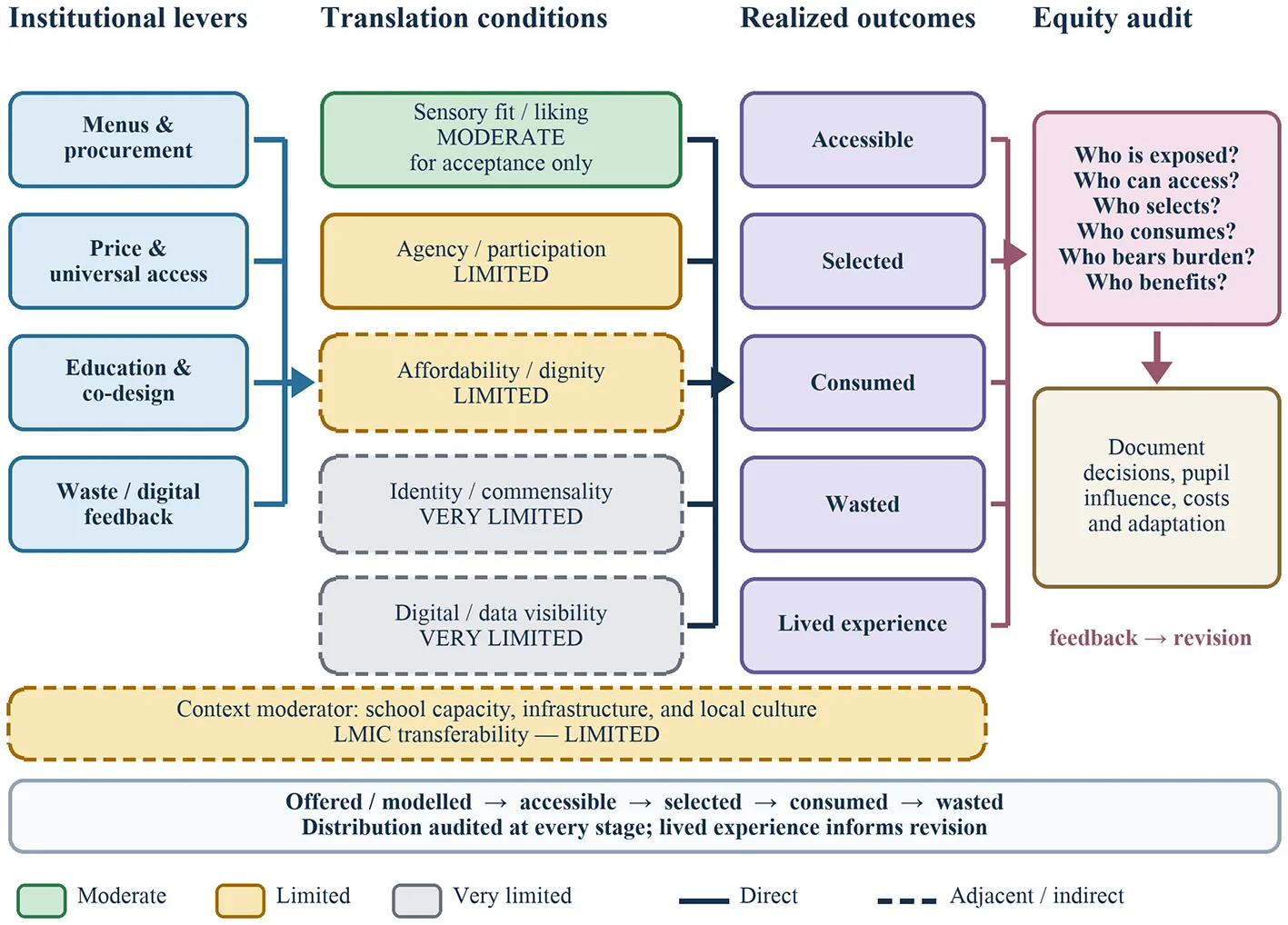
Evidence-coded provision-to-practice framework for realized sustainability in school food environments.

Figure 2 maps narrative relative support; Table 1 presents study-level country, sample, design, follow-up, evidence level, outcome type, source availability, and appraisal completeness. The synthesis used three categories: Moderate, Limited, and Very limited. Sensory liking and neophobia had Moderate support for liking/ acceptance only; menu reform, waste feedback, education/information, agency/participation, affordability/ universal- meal governance, and lower- and middle-income country transferability were Limited. Identity/recognition/commensality and digital influence were Very limited for downstream outcomes. Fifteen of 25 publications had at least one applicable appraisal domain judged No, and 13 had at least one domain judged Cannot tell; these judgments bounded pathway claims rather than being converted into a total score. Figure 2 fill color denotes pathway-level narrative relative support across independent study families. Border style indicates whether a pathway includes evidence at a Direct level or only Adjacent/indirect evidence; it does not assign the same directness to every downstream outcome. The downstream boxes are conceptual endpoints, and study-specific evidence-to-outcome links are reported in Table 1. Blue boxes identify institutional levers and are not a fourth support category.

### 3.2 Menus, procurement, and sensory acceptability

Menu reform changes both environmental exposure and the meal pupils encounter. In six Swedish schools, a 20% lower-carbon menu did not reduce consumption, increase plate waste, or lower satisfaction over seven weeks (12). In three others, a four-week optimized food list reduced modeled greenhouse-gas emissions by 40% and cost by 11% without significant short-term differences in consumption, waste, or satisfaction (13). Linked qualitative evidence identified staff involvement, leadership, kitchen capacity, and adaptation as implementation conditions (14). These findings support short-term local acceptance, not long-term equivalence or transferability.

In Ghana, affordable modeled menus diverged from existing food baskets, and animal-source-food constraints increased modeled environmental impact (15). A Kenyan model examined underutilized species but did not test implementation or acceptance (16). Sensory findings were more consistent: Italian studies linked neophobia, familiarity, emotion, household diet, education, and appeal to reported acceptance (17, 18); French citywide ratings found similar liking for vegetarian and nonvegetarian meals without measuring intake (19); and European testing associated liking with lower **self-reported** waste (20). South African and Amazonian evidence emphasized familiarity, preparation, infrastructure, and biodiversity (21, 22), while a parent norm message changed menu choice differently across education groups (23). The implication is local recipe testing and recognizable formats, not a universal menu.

### 3.3 Waste feedback, education, and digital influence

Food waste makes the provision–practice gap observable. Across Austria, Germany, and Sweden, service-level plate-waste tracking was associated with a 17% reduction from a 23-g baseline for up to 15 months, whereas pedagogic meals and workshops showed no clear change (24). A two-school US curriculum plus peer campaign reported lower vegetable waste at 5 months, although baseline differences weakened attribution (25). A randomized classroom intervention did not change selection or waste, and longitudinal Italian education effects were not consistently sustained (26, 27). A multi-component English program operated for 18–24 months but partly used pupil-reported intake (28). Within these design limits, operational feedback was associated with more favorable measured waste outcomes than information-only approaches, but the heterogeneous evidence does not establish comparative causal superiority.

Digital evidence remained narrow. The tracker study examined service-level data feedback rather than a consumer platform (24). In an exploratory recipe comparison, children preferred child- and cook-designed carrot-peel pies to a ChatGPT-generated version because texture and unfamiliar taste undermined the latter (29). These studies do not establish general effects of artificial intelligence, apps, digital payment, recommendation systems, or social media. Digital tools should be evaluated as food-environment actors whose access, privacy, commercial incentives, cultural assumptions, and non-digital alternatives may alter participation and equity.

### 3.4 Student agency, co-design, and institutional power

Agency describes what pupils can do within rules governing menus, budgets, portions, payment, time, and infrastructure. German transformation research documented tensions among inclusion, sustainability, staff capacity, and student participation rather than a linear implementation process (30). In the United Kingdom, pupils using a free-meal allowance described price, fingerprint payment, taste, availability, and limited sustainable options as interacting constraints (31). Qualitative OPTIMAT findings likewise showed that menu change depended on organizational authority and kitchen implementation, not pupil preference alone (14). A parent social-norm experiment demonstrated that choice architecture can change selections, but education-related heterogeneity cautions against assuming equal responsiveness (23).

These findings distinguish formal choice from realized influence. Participation is consequential when pupils can identify problems, test dishes, interpret waste data, affect decisions, and revisit outcomes. Current evidence seldom documents transferred authority or durable effects on intake and waste. Evaluations should therefore record who controls menus and budgets, which proposals are adopted, how constraints are explained, and whether feedback produces observable revision.

Developmental stage conditions agency: parents mediate many primary-school choices, younger children contribute sensory and service experience, and adolescents more often encounter purchasing autonomy, peers, and visible payment rules. The evidence did not support robust age-stratified pathway ratings. Future evaluations should report age or grade and respondent unit explicitly and test whether decision rights and translation barriers change across development.

### 3.5 Cultural identity, commensality, and recognition

School lunch is a public social practice. South African mixed-methods evidence linked sorghum acceptance and waste to cultural familiarity, preparation, and serving conditions (22); a Brazilian Amazon journal-supplement abstract contrasted local biodiversity with ultra-processed provision (21); and Chilean food handlers identified familiarity, sensory rejection, and implementation constraints in canteen waste (9). UK pupils also described how payment systems and restricted options made price and status visible (31). These studies explain why an objectively sustainable option may still be avoided.

Religion, migration, disability, and culturally equivalent accommodation remained largely unmeasured. This is a core equity gap because menu names, service lines, restricted choice, sensory unfamiliarity, and unequal accommodation can determine whether pupils feel recognized or marked as different. Cultural acceptability should be measured through dietary accommodation, perceived recognition, social safety, meal equivalence, and participation—not inferred only from deviation from existing food baskets or average liking.

### 3.6 Affordability, universal meals, and distributional equity

Affordability operates as both a material constraint and a social signal. UK pupils described a free-meal allowance that did not reliably purchase a meal perceived as healthy, tasty, and sustainable (31). Interviews with SNAP-eligible US adolescents linked plate waste to food quality, social influence, policy rules, and limited control (32). A universal-breakfast study similarly found that palatability, logistics, and surplus could generate waste within an access-promoting program (33). These mechanisms show why nominal entitlement does not ensure an acceptable or low-waste meal.

New Zealand and global program-level analyses added direct environmental dimensions to universal or large-scale provision by considering food or packaging waste, procurement, local sourcing, and equity (34, 35). Their unit of analysis, however, was the program rather than individual child selection and intake. Evidence therefore supports integration of sustainability and equity at governance level, while child-level causal effects remain very limited. Universal provision should be evaluated through meal quality, reliable availability, non-segregated service, participation, intake, waste, and subgroup experience rather than enrollment alone.

### 3.7 Lower- and middle-income country transferability

The evidence base does not support direct transfer of European menu, kitchen, or digital interventions to lower-resource systems. Ghanaian optimization exposed tensions among nutrient adequacy, affordability, existing foods, and environmental impact without implementation or tasting (15). Kenyan modeling examined underutilized species in an informal-settlement program but did not establish uptake (16). A Brazilian Amazon journal-supplement abstract and studies from South Africa and Chile added biodiversity, cultural familiarity, serving infrastructure, and staff implementation evidence (9, 21, 22). None jointly evaluated environmental impact, nutritional adequacy, child intake, waste, cost, and distribution over time.

Transfer should begin with local staples, culinary knowledge, religious and cultural diversity, seasonal supply, and school/vendor capacity. Safe water, refrigeration, energy, storage, transport, equipment, staffing, and reliable procurement may determine which menu is feasible before acceptance is tested. Multi-criteria planning should protect nutrition and food safety while comparing environmental impact, cost, cultural fit, workload, and distributional access. Locally generated alternatives and implementation resources require evaluation rather than treating high-income-country packages as context-free technologies.

## 4 Discussion

### 4.1 What the framework changes

The main conceptual contribution is to replace the offered menu as the endpoint with a provision–access/selection–intake–waste pathway and a cross-cutting equity audit. Institutions control procurement, prices, service, time, information, and decision rights; pupils encounter these arrangements through taste, identity, peer relations, affordability, and practical agency. At every stage, evaluation asks who is exposed, who can access and select, who consumes, who bears stigma or burden, and who receives nutritional and environmental benefit. A lower-impact meal that is unavailable, unaffordable, culturally alienating, uneaten, or discarded has not achieved realized sustainability. Conversely, a popular or equitable meal cannot be called sustainable without environmental measurement.

This framework changes design, evaluation, and governance. **Design** should set nutrition and food-safety floors, environmental targets, and budget and infrastructure constraints, then co-design culturally recognizable alternatives within those boundaries. **Evaluation** should measure provision, selection, intake, waste, lived experience, and subgroup distribution as distinct outcomes. **Governance** should document who decides, how pupil input changes menus or service, what resources implementation requires, and how feedback triggers revision. Schools should use iterative menu portfolios rather than one-off acceptance testing.

The chain also locates implementation failure. Low participation may indicate affordability or dignity barriers; high waste may reflect recipe, portion, time, or service mismatch. Schools can combine kitchen indicators with pupil recognition and subgroup participation. A dashboard should pair environmental intensity per meal offered and consumed with nutrient adequacy, cost, participation, edible waste, and unequal burden, preventing improvement on one metric from concealing poorer intake or exclusion. Pupil councils and food-service teams can review these data, test a bounded change, document resources and adoption, and retain or reverse it using nutritional, environmental, experiential, and distributional results. Student voice thereby becomes accountable implementation rather than one-off consultation.

### 4.2 Balancing health, cost, environment, culture, and equity

Decision makers need explicit multi-criteria rules. Plant-forward reform may reduce modeled emissions yet miss nutrient, familiarity, or participation targets; waste reduction may conserve resources without improving diet quality. A practical sequence is to: (1) set nutrition, safety, portion, and affordability thresholds; (2) compare feasible portfolios on environment, cost, infrastructure, and cultural fit; (3) test selection, intake, and waste in routine service; (4) examine socioeconomic, disability, religion, migration, and school-capacity differences; and (5) revise procurement and recipes transparently.

Trade-offs should be reported rather than averaged away. Distributional checks are essential when lower prices, smaller portions, restricted choice, or culturally narrow menus transfer burdens to particular pupils or schools. Environmental performance, health, and equity are joint decision criteria, while pupil experience supplies evidence about whether the selected portfolio works in practice.

### 4.3 Overall evidence strength, bias, and contradictory findings

Except for Moderate relative support for sensory liking and neophobia on acceptance outcomes, pathway support is Limited to Very limited. Within the focused set, menu-reform and waste-feedback families included the largest share of observed outcomes, but nonrandom assignment, few sites, and limited follow-up constrain causal interpretation. Sensory studies are often cross-sectional or rating-based. Co-design, identity, and commensality evidence is mainly qualitative or pilot-based. Child-level universal-meal studies omit environmental outcomes; program studies do not jointly measure child intake. Modeling clarifies trade-offs but cannot establish acceptance or intake.

Contradictory and null findings prevent simple intervention rankings. Lower-carbon Swedish menus reduced modeled impact without measurable short-term loss of acceptance, while additional information produced no independent benefit (12, 13). The randomized classroom trial found no significant selection or plate-waste effect, and the longitudinal education study found changes that were not consistently sustained (26, 27). The waste tracker was associated with lower waste while pedagogic meals and workshops were not (24). Program-level universal-meal evidence linked equity with procurement and waste, yet qualitative studies also identified palatability, logistics, and social constraints (32–35). These patterns support mechanism-specific, context-sensitive evaluation rather than assuming that “education,” “universal provision,” or “plant-forward menus” have uniform effects.

Selection and publication bias remain plausible. English-indexed Scopus and Web of Science coverage may omit records without English titles or abstracts, as well as gray, program, and government evidence, particularly from lower- and middle-income countries. Reference chaining can favor well-cited networks, and positive pilots may be more publishable than failed implementation. Heterogeneous outcomes and designs made funnel plots and pooled effects inappropriate. To make these risks inspectable, we retain the non-binding AI-prioritization ledger, report the author-verified 85-row source ledger, provide row-level sampling bases and study-family grouping, distinguish full-text from source-limited appraisal, retain null findings, and report coverage sensitivity for all 46 eligible non-sampled publications. These safeguards do not make the review exhaustive or eliminate residual selection bias.

### 4.4 Prioritized research agenda

Three priorities follow. **First, immediately feasible studies** should add a minimum outcome set—offered menu profile, selection, intake, plate waste, satisfaction, and subgroup participation—to ongoing school-meal programs, together with kitchen, staffing, procurement, time, and cost data. **Second, near-term comparative evaluations** should test culturally adapted menu portfolios and meaningful pupil decision roles across schools with different resources, using repeated measures and objective outcomes. **Third, longer-term system research** should evaluate environmental, nutritional, financial, cultural, and distributional outcomes together across policy transitions, especially in lower- and middle-income countries and among pupils differentiated by disability, religion, migration, and socioeconomic position.

Digital research should be embedded within these priorities rather than treated as a separate solution class. Evaluations should compare non-digital alternatives, disclose commercial interests, assess privacy and access burdens, and audit whether tools improve institutional responsiveness without excluding pupils or encoding narrow cultural preferences.

## 5 Conclusion

Researchers should evaluate the complete provision–access/selection–intake–waste pathway, audit distribution at every stage, group linked publications, and match claims to study design and relative support. Schools should co-design within explicit nutrition, safety, environmental, cost, and infrastructure constraints, then use routine feedback to revise menus and service. Policymakers should fund implementation capacity and require distributional reporting rather than judging success from procurement standards alone. Sustainable school food is realized when a lower-impact, nutritious meal becomes accessible, culturally recognizable, socially acceptable, consumed with dignity, and equitably supported across pupils and schools.
